# Genome-Wide Identification of the bHLH Gene Family in *Rhododendron delavayi* and Its Expression Analysis in Different Floral Tissues

**DOI:** 10.3390/genes15101256

**Published:** 2024-09-26

**Authors:** Jian Dong, Ya-Wen Wu, Yan Dong, Ran Pu, Xue-Jiao Li, Ying-Min Lyu, Tian Bai, Jing-Li Zhang

**Affiliations:** 1 College of Landscape and Horticulture, Yunnan Agricultural University, Kunming 650201, China; dongjian970202@163.com (J.D.); yawenwu@ynau.edu.cn (Y.-W.W.); puran1026@163.com (R.P.); lixuejiao@ynau.edu.cn (X.-J.L.); 2 China Flower Association, Beijing 100102, China; fswallow0609@163.com; 3 School of Landscape Architecture, Beijing Forestry University, Beijing 100083, China; luyingmin@bjfu.edu.cn; 4 National Rhododendron Germplasm Resource Bank, Kunming 650201, China

**Keywords:** *Rhododendron delavayi* Franch, bHLH gene family, expression pattern, floral tissues

## Abstract

Background: The bHLH genes play a crucial role in plant growth, development, and stress responses. However, there is currently limited research on bHLH genes in the important horticultural plant *Rhododendron delavayi* Franch. Methods: In this study, we conducted a comprehensive genome-wide identification and in-depth analysis of the bHLH gene family in *R. delavayi* using bioinformatics approaches. Results: A total of 145 bHLH family members were identified, encoding proteins ranging from 98 to 3300 amino acids in length, with molecular weights ranging from 11.44 to 370.51 kDa and isoelectric points ranging from 4.22 to 10.80. These 145 bHLH genes were unevenly distributed across 13 chromosomes, with three bHLH genes located on contig 52. Chromosome 8 contained the highest number of bHLH family members with 19 genes, while chromosomes 9 and 13 had the lowest, with 7 genes each. Phylogenetic analysis revealed a close evolutionary relationship between bHLH genes in *R. delavayi* and *Arabidopsis thaliana*. Subcellular localization analysis indicated that most bHLH genes were located in the nucleus. Promoter analysis of *R. delavayi* bHLH genes revealed the presence of various cis-regulatory elements associated with light responses, methyl jasmonate responses, low-temperature responses, and coenzyme responses, suggesting that bHLH genes are involved in multiple biological processes in *R. delavayi*. Through transcriptome analysis, we identified three key functional genes—*Rhdel02G0041700*, *Rhdel03G0013600*, and *Rhdel03G0341200*—that may regulate flower color in *R. delavayi*. Conclusions: In conclusion, our study comprehensively identified and analyzed the bHLH gene family in *R. delavayi* and identified three bHLH genes related to flower color, providing a foundation for molecular biology research and breeding in *R. delavayi*.

## 1. Introduction

*Rhododendron* L. belongs to the family Ericaceae and encompasses evergreen, deciduous, or semi-deciduous shrubs or trees. It is regarded as one of China’s top ten traditional flowers and, along with gentians (*Gentiana*) and primroses (*Primula*), is recognized as one of “China’s top three natural flowers” and “the world’s top three alpine flowers”. *Rhododendron* plays a crucial role in some of the world’s most fragile ecosystems, particularly in subtropical broad-leaved evergreen forests and the ecosystems of the Himalayas and Hengduanshan regions, where it contributes significantly to slope stabilization and watershed protection. *Rhododendron* species are integral components of natural ecosystems in alpine and subalpine scrublands, coniferous forests, mixed coniferous–broadleaf forests, and broad-leaved evergreen forests in southwestern China. Their ecological importance is underscored by their contribution to the stability of these ecosystems. Additionally, *Rhododendron* is of considerable interest in research on population biology, flora, and biodiversity due to its high ecological value. It also holds significant potential as a horticultural resource, medicinal herb, and industrial raw material. China possesses a rich diversity of *Rhododendron* species, with over 1000 species known worldwide [1], approximately 542 of which are found in China [2]. Yunnan province is particularly rich in *Rhododendron* diversity, with around 320 species [3]. These plants are widely admired for their vibrant colors, elegant flower forms, and upright growth habits, making them highly valuable for ornamental use in gardens, scenic areas, and indoor environments [4].

*R.delavayi* is an evergreen shrub or small tree within the *Rhododendron* genus of the Ericaceae family. It typically grows in evergreen broad-leaved forests or shrub thickets at altitudes ranging from 1200 to 3200 m [5,6,7]. This species is notable for its high aesthetic, medicinal, and utilitarian value. However, its strict habitat requirements, particularly in terms of water availability, pose challenges to its widespread use in landscaping applications [8,9]. With the closure of forests and over-excavation, wild *Rhododendron* resources are gradually decreasing. The rapid breeding of high-quality *Rhododendron* seedlings is an urgent problem for the protection and utilization of *Rhododendron* resources. From the point of view of industrialization and development of *Rhododendron*, the goal of breeding should be especially devoted to solving the difficulties in cultivation, such as alkali resistance, heat resistance, cold resistance, and disease resistance. *Rhododendrons* thrive in acidic soils, with neutral or alkaline soils being a major constraint on their widespread application. In landscape gardening, *Rhododendrons* are typically cultivated in open fields over large areas with relatively extensive management. Due to significant annual temperature fluctuations and increased risk of cross-infection by pests and diseases, only a limited number of *Rhododendron* species are suitable for use as ornamental trees. From a breeding perspective, the development of new varieties with tolerance to alkaline soils (particularly root and stem tolerance), heat, cold, and disease resistance has long been a central goal in modern *Rhododendron* breeding programs. The selection and breeding of alkali-resistant (rootstock), heat-resistant, cold-resistant, and disease-resistant varieties has always been one of the objectives of modern *Rhododendron* breeding. Wild *Rhododendrons* are mainly shrubs and small trees. When selecting and breeding plants, it is easier to cultivate good progeny by taking the beautiful tree *Rhododendron* or dwarf *Rhododendron,* suitable for pot planting, as the breeding parent. Many *Rhododendrons* have natural fragrance when the flowers bloom, which carries for miles.

The bHLH gene family is widely distributed across animals, plants, and microorganisms [10]. It is characterized by a basic-helix-loop-helix structure consisting of approximately 60 amino acids, comprising a basic region and an HLH region primarily involved in DNA binding [11,12]. The overexpression of *bHLH122* in *Arabidopsis* has been shown to significantly enhance plant stress resistance [13]. In chrysanthemum mutants involved in anthocyanin glycoside synthesis, the downregulation of the regulatory genes *MYB* and *bHLH* is closely associated with the development of white flowers [14]. In the aerial stems of *Panax notoginseng*, the MBW complex, composed of *bHLH*, *MYB*, and *WD40*, contributes differentially to the synthesis of color glycosides by regulating the expression of various structural genes [15].

*R.delavayi*, known for its vibrant flower colors and extended flowering period, adds striking springtime hues to gardens and parks. Its large, densely clustered flowers enhance visual impact and overall ornamental appeal. bHLH transcription factors play a crucial role in regulating flower color. However, there are fewer reports on the study of bHLH in *R. delavayi*. In this study, we identified bHLH transcription factors, analyzed the structural characteristics and physicochemical properties of this family through bioinformatic analysis, and analyzed the expression of different flower colors of *R. delavayi*, so as to lay a theoretical foundation and reference for the in-depth study of the bHLH transcription factor family. Using the high-quality genome of *R. delavayi*, this study conducted a comprehensive genome-wide identification and analysis of the bHLH gene family. A total of 145 bHLH genes were identified, and their distribution, physicochemical properties, and expression patterns were systematically examined. The findings suggest that three specific bHLH genes may play a critical role in regulating flower color in this species.

## 2. Materials and Methods

### 2.1. Materials

The genomic sequence and GFF annotation file of *R. delavayi* are derived from the *Rhododendron* Plant Genomic Database (http://bioinfor.kib.ac.cn/RPGD/index.html). Gffread (Version 0.12.7) was used to extract protein sequences from the genome, and a Perl script was employed to extract the longest transcript of each gene for analysis. The genomic data and gene family classification information of the model plant *A. thaliana* were obtained from the TAIR database (https://www.arabidopsis.org/) [16]. The transcriptome data of *R. delavayi* flower color is sourced from a study by Fenfang Long et al. [17].

### 2.2. Methods

#### 2.2.1. Identification of the Complete Genome of bHLH Genes in *R. delavayi*, Prediction of Physicochemical Properties of Encoded Proteins, and Analysis of Gene Locus Prediction

The HMM file (PF00010) of the bHLH transcription factor family was downloaded from Pfam. The HMMER (Version 3.3.1) [18] was utilized to identify the bHLH gene family in *R. delavayi*. The HMMER output was filtered using a threshold of 1 × 10^−5^. The filtered domain sequences were aligned using clustalw (Version 2.1), and the aligned results were used to build a new HMM file using hmmbuild with default parameters. The newly constructed HMM file was then employed with HMMER for whole-genome identification of bHLH genes in *R. delavayi*, with a threshold of 0.001 used for result filtering. The protein sequences of the filtered genes were verified using NCBI-CDD with threshold 0.01, and the sequences containing bHLH were identified as the final set of bHLH genes.

The identified members of the bHLH gene family in *R. delavayi* were analyzed by using the following website, https://www.expasy.org, to determine the protein length, molecular weight, and isoelectric point. The subcellular localization prediction was conducted using Wolfpsort (https://wolfpsort.hgc.jp/).

#### 2.2.2. Phylogenetic and Evolutionary Analysis of the *R. delavayi* bHLH Gene Family

The protein sequences of the bHLH gene family in *R. delavayi* and the bHLH gene family in *A. thaliana* were aligned using mafft (Version 7.515) with default parameters [19]. Multiple sequence alignment was performed for the full-length protein sequences encoded by all members of the bHLH gene family. The maximum likelihood method was used to construct a phylogenetic tree using FastTree software (Version 2.1.11) with 1000 repetitions [20]. The phylogenetic tree was processed using the ggtree package (Version 3.12.0) in R [21].

#### 2.2.3. Structural Analysis of Genes in the *R. delavayi* bHLH Gene Family

The annotation information of the members of the bHLH gene family in *R. delavayi* was extracted from the GFF3 annotation file of the *R. delavayi* genome. The gene structure of the bHLH gene family in *R. delavayi* was visualized using GSDS 2.0 (https://gsds.gao-lab.org/) [22].

#### 2.2.4. Conserved Motif Analysis of *R. delavayi* bHLH Gene Family Proteins

The MEME software (Version 5.5.2) (http://meme-suite.org/tools/meme) was used to analyze the conserved motifs in the protein sequences of the bHLH gene family in *R. delavayi* [23]. The analysis was performed using the anr mode with 10 motifs and a sequence recognition length of 6–100. The remaining parameters were set to default values. The output results from MEME were visualized using the ggmotif package (Version 0.2.1) in R [24].

#### 2.2.5. Promoter Analysis of the *R. delavayi* bHLH Genes

A Perl script was used to extract the upstream 1500 bp region of the promoter sequences of the bHLH gene family from the whole-genome sequence of *R. delavayi*. The extracted sequences were then analyzed using the PlantCARE database (https://bioinformatics.psb.ugent.be/webtools/plantcare/html/) to determine the types and functions of cis-acting elements [25]. The predicted results were visualized using the ggplot2 package (Version 3.5.1) in R [26].

#### 2.2.6. Expression Pattern Analysis of the *R. delavayi* bHLH Gene Family

In order to explore the potential functions of the bHLH gene family in *R. delavayi* in flower color formation, we downloaded the original RNA-Seq data related to flower color from the NCBI database with the data ID PRJNA907866 [17]. Firstly, we used HISAT2 (Version 2.2.1) to align the transcriptome data to the *R. delavayi* genome [17] with default parameters. Then, we converted the SAM files to BAM files using samtools (Version 1.6) [27]. StringTie (Version 2.2.1) was used to calculate the gene expression levels, and the FPKM values from the output results were used for subsequent differential analysis [28]. Differential expression genes were identified using the R package DESeq2 (Version 1.44.0) [29]. Genes with a *q*-value < 0.05 and |log_2_FoldChange| > 1 were selected as significantly differentially expressed genes. Among them, genes with a log_2_FoldChange > 1 were considered significantly upregulated, while genes with a log_2_FoldChange < −1 were considered significantly downregulated.

## 3. Result and Analysis

### 3.1. Identification and Physicochemical Properties of R. delavayi bHLH Gene Family Proteins

**Figure 1:**
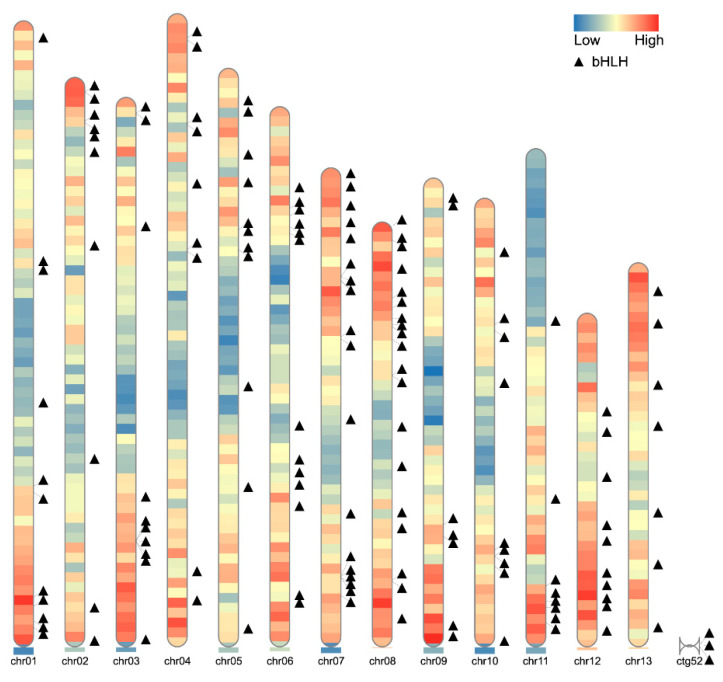
Chromosomal localization of the members of the bHLH gene family of *R. delavayi*.

A total of 145 bHLH gene family members were identified in *R. delavayi* using an HMMER search (Supplementary Table S1). These genes are unevenly distributed across 13 chromosomes, with most concentrated near the chromosome ends. Specifically, 19 bHLH genes are located on chromosome 8, 7 on chromosomes 9 and 13, and 3 on ctg52 (Figure 1).

The location of the bHLH gene family members on the chromosome of *R. delavayi* can be seen through Figure 1. Figure 1 reveals that most of the bHLH gene family members are concentrated at both ends of the chromosome. Among them, we also found a more interesting place where three bHLH gene family members were distributed on ctg52.

**Figure 2:**
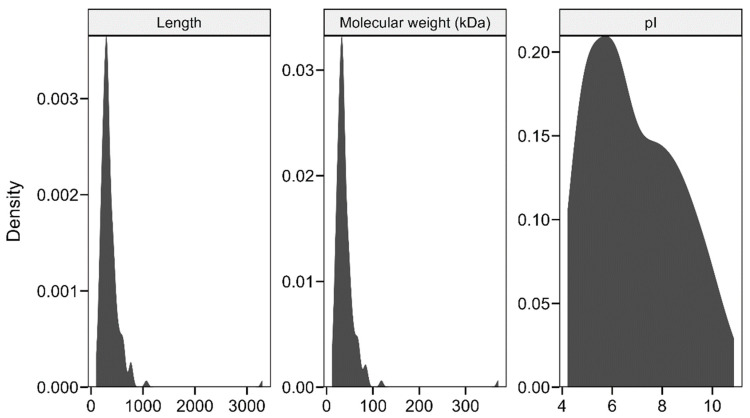
Physicochemical properties of encoded proteins.

The proteins encoded by these 145 bHLH gene family members vary in length from 98 to 3300 amino acids (Figure 2), with an average length of 364.65 amino acids. Their molecular weights range from 11.44 to 370.51 kDa, with an average of 40.44 kDa. The longest and heaviest protein is encoded by Rhdel05G0030600, whereas the shortest protein, with a molecular weight of 11.44 kDa, is encoded by Rhdel10G0216600. The isoelectric points of these proteins range from 4.22 to 10.87, with an average of 6.86. Of the 145 genes, 83 have isoelectric points below 7, while 62 have isoelectric points above 7.

The physicochemical properties of the proteins encoded by the bHLH gene family of *R. delavayi* are shown in Figure 1. 145 bHLH gene families encoded proteins with lengths ranging from 98 to 3300 amino acids, with an average encoded protein length of 364.65 amino acids, molecular weights ranging from 11.44 to 370.51 kD, with an average of 40.44 kD, and isoelectric ranges from 4.22 to 10.87 with an average value of 6.86. A total of 83 genes had an isoelectric point less than 7, and another 62 bHLH genes had an isoelectric point greater than 7.

### 3.2. Phylogenetic Analysis of the R. delavayi bHLH Gene Family

**Figure 3:**
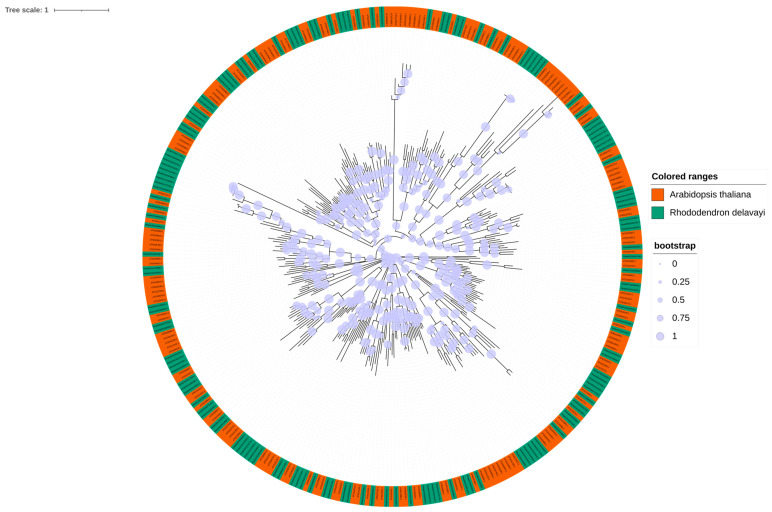
Phylogenetic developmental tree of bHLH family in *R. delavayi*.

A phylogenetic tree was constructed to elucidate the evolutionary relationships between the members of the *R. delavayi* bHLH gene family using the protein sequences of the filtered *R. delavayi* bHLH family proteins and randomly selected protein sequences of *A. thaliana* (L.) Heynh bHLH genes. As illustrated in (Figure 3), the distribution of bHLH gene family members is relatively uniform in *Arabidopsis* and *R. delavayi*.

Phylogenetic tree analysis of the protein sequences of the bHLH family proteins of *R. delavayi* and a randomly selected protein sequence of the *Arabidopsis* (*A. thaliana* (L.) Heynh) bHLH gene showed good homology.

### 3.3. Gene Structure Analysis of the R. delavayi bHLH Gene Family

**Figure 4:**
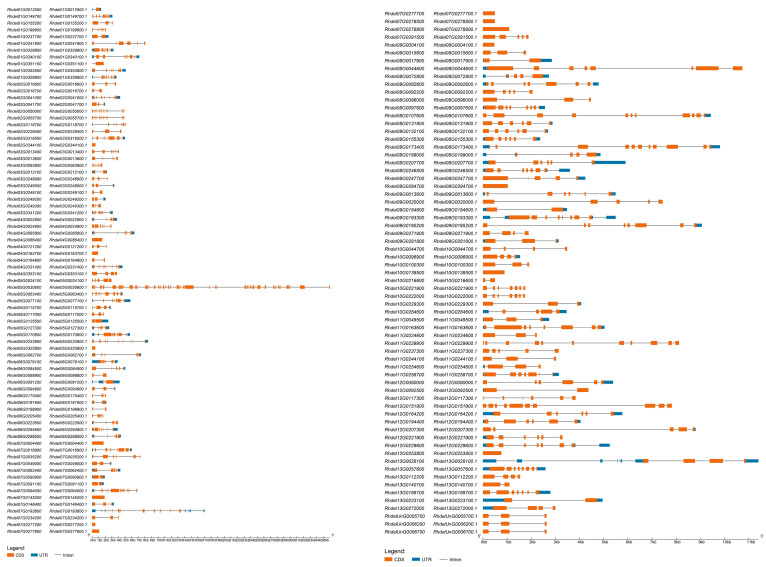
Gene structure of members of the bHLH gene family of the *R. delavayi*.

Gene structure analysis of the *R. delavayi* bHLH gene family revealed that most genes in the family contain introns (Figure 4). Notably, 14 genes, including *Rhdel02G0344100, Rhdel04G0086400, Rhdel04G0163700, Rhdel05G0325800, Rhdel07G0004400, Rhdel07G0145200, Rhdel07G0277200, Rhdel07G0277600, Rhdel07G0278500, Rhdel07G0278800, Rhdel08G0284700, Rhdel10G0138500*, and *Rhdel12G0253800*, are intronless. The gene with the longest sequence, *Rhdel05G0030600*, harbors 42 introns.

The gene structure of the members of the bHLH gene family of *R. delavayi* shows that most of the *R. delavayi* s bHLH gene family contains introns. Among them, the longest sequence is Rhdel05G0030600 gene, containing 42 introns.

### 3.4. Conserved Motif Analysis of the R. delavayi bHLH Gene Family

**Figure 5:**
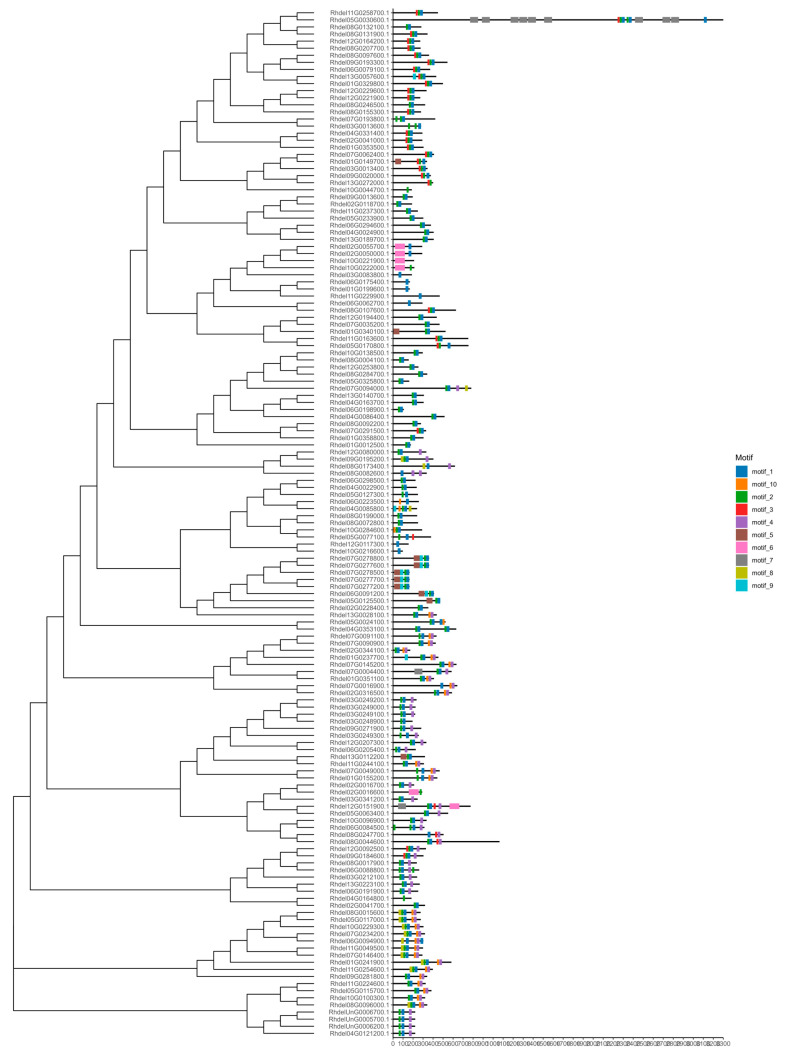
Motif analysis of conserved motifs of proteins encoded by members of the bHLH gene family of *R. delavayi*.

A total of 10 conserved protein motifs, designated Motif1 to Motif10, were identified across the 145 bHLH gene family members, exhibiting a largely consistent distribution pattern (Figure 5). Both Motif1 and Motif2 were present in all 145 *R. delavayi* bHLH gene family members. Motif2 appeared twice in the proteins encoded by *Rhdel07G0193800* and *Rhdel03G0013600*. The protein encoded by Rhdel05G0030600, the longest protein in the family, harbored 9 instances of Motif7, 3 instances of Motif1, 2 instances of Motif2, and 1 instance of Motif3. The proteins encoded by *Rhdel10G0044700, Rhdel03G0083800, Rhdel06G0175400, Rhdel01G0199600, Rhdel11G229900*, and *Rhdel06G0062700* each contained only 1 instance of Motif1. The proteins encoded by *Rhdel10G0044700* and *Rhdel04G0164800* each contained only 1 instance of Motif2.

Motif analysis of conserved motifs of proteins encoded by members of the bHLH gene family of *R. delavayi* detected a total of 10 protein conserved motifs with essentially the same distribution in 145 bHLH gene family members. Motif1 and Motif2 were both present. A total of 2 occurrences were observed on proteins encoded by Rhdel07G0193800 and Rhdel03G0013600 of Motif2. The protein encoded by Rhdel05G0030600 was the longest, with 9 occurrences of Motif7, 3 occurrences of Motif1, 2 occurrences of Motif2, and 1 occurrence of Motif3 on it.

**Figure 6:**
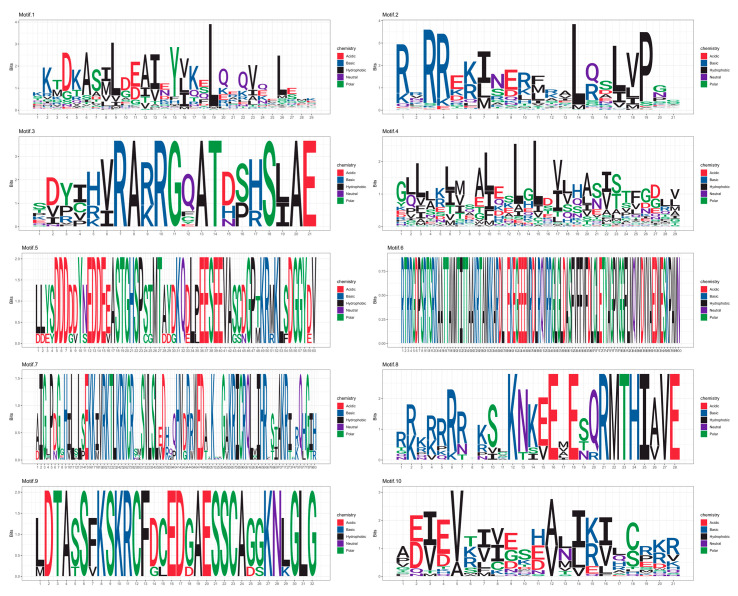
MEME Predicted 10 Motifs for the RdbHLH Transcription Factor.

Analysis of the Motif sequences revealed the sequence information of Motif1 to Motif10, as depicted in the Motif logo diagrams (Figure 6).

The 10 Motifs of RdbHLH transcription factor were predicted using MEME software. The sequence information of Motif1~Motif10 can be known by this figure.

### 3.5. Cis-Regulatory Element Analysis of R. delavayi bHLH Gene Family Promoters

**Figure 7:**
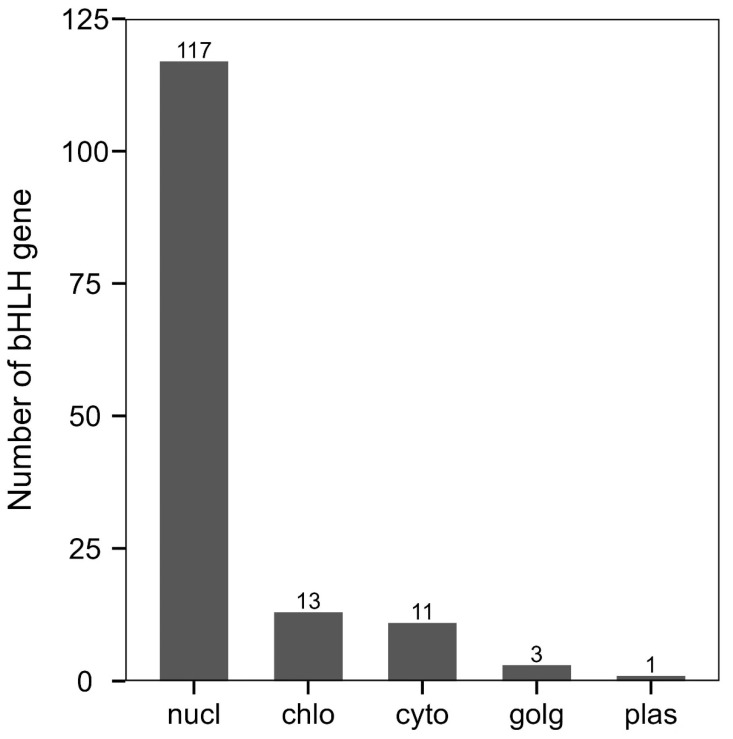
Prediction of subcellular localization of bHLH gene family members in *R. delavayi*.

Subcellular localization analysis revealed that the majority of bHLH gene family members (117) were localized to the nucleus, while only one bHLH gene family member was localized to the plasma membrane (Figure 7).

The content of bHLH gene family members in different locations was analyzed by subcellular localization. bHLH gene family members were found to be distributed in nucl, chlo, cyto, golg, and plas. nucl had the highest distribution of bHLH gene family members while plas contained the lowest number of bHLH gene family members.

**Figure 8:**
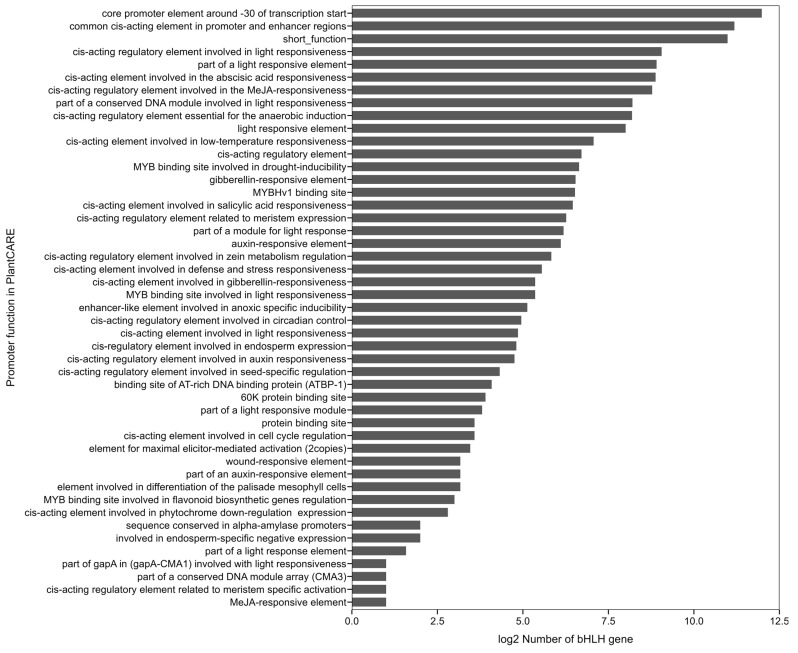
Results of promoter function analysis.

Cis-regulatory element analysis was performed on the sequences 1500 bp upstream of the translation start sites of *R. delavayi* bHLH gene family members (Figure 8). A total of 47 cis-regulatory elements were identified, encompassing various response pathways, including light response, methyl jasmonate response, low-temperature response, and coenzyme response. The analysis demonstrated that the distribution of core promoters in the vicinity of the transcription start site −30 was most prominent among *R. delavayi* bHLH gene family members, whereas their distribution in methyl jasmonate response was less pronounced. There are more members of cis-acting elements associated with the light response, i.e., members of the bHLH gene family are more closely related to the light response, but among the many cis-acting elements, the core promoter near the transcriptional start point −30 is the most closely related.

Cis-acting elements were analyzed for the sequence 1500 bp upstream of the translation initiation site for members of the bHLH gene family of *R. delavayi*. A total of 47 cis-acting elements were detected, involving various reaction processes such as light reaction, methyl jasmonate reaction, low temperature reaction, coenzyme reaction, etc.

### 3.6. Expression Pattern Analysis of R. delavayi bHLH Gene Family Members

**Figure 9:**
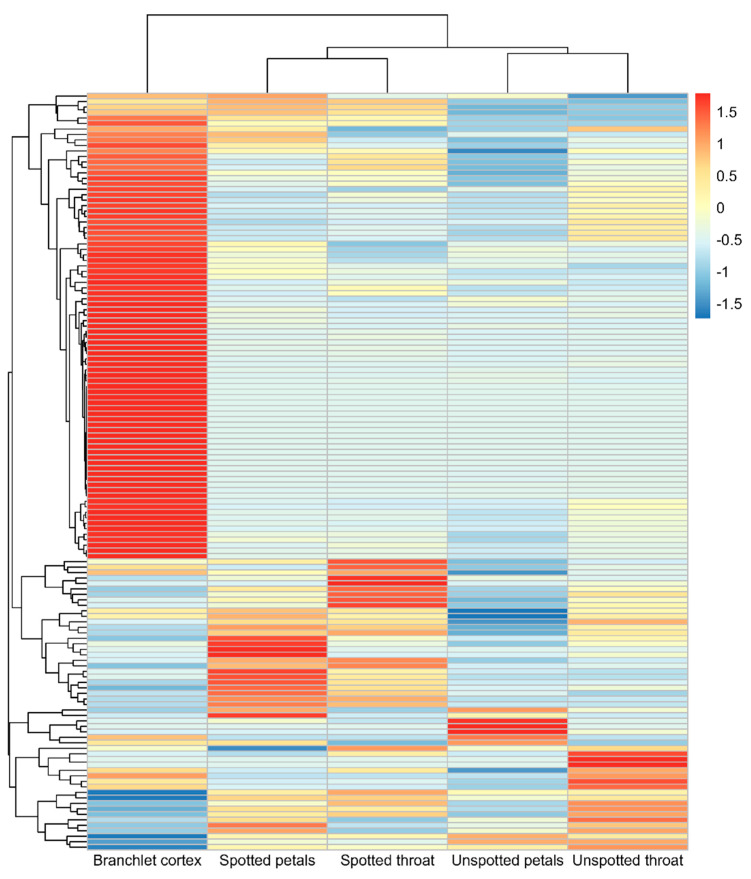
Expression patterns of the bHLH gene of *R. delavayi* in five flower-colored plants.

**Figure 10:**
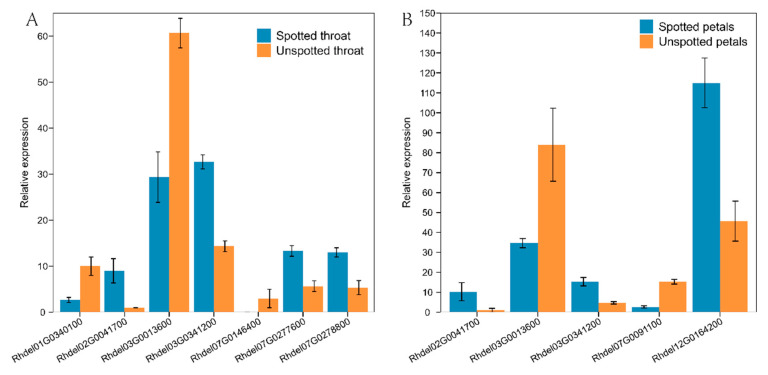
Differentially expressed gene. (**A**) shows the expression in calyx and (**B**) shows the expression in Petals.

Transcriptome analysis revealed that the expression patterns of *R. delavayi* bHLH genes exhibited specificity among five flower color variants (Figure 9). Differential expression analysis of the transcriptome data (Figure 10) indicated that the highest expression levels were observed in Branchlet cortex, while Unspotted petals exhibited the lowest relative expression levels. These findings suggest that *Rhdel02G0041700, Rhdel03G0013600*, and *Rhdel03G0341200* may be key genes involved in regulating flower color in *R. delavayi*.

Expression patterns of the bHLH gene of *R. delavayi* in five flower-colored plants were analyzed by transcriptomic data. The bHLH gene of *R. delavayi* showed specificity in the five flower-colored plants. It was highly expressed in Branchlet cortex and least expressed in Unspotted petals. The throat in this Figure is the calyx portion of the floral organ.

By analyzing the differentially expressed genes in the transcriptome, it is possible to find the key genes among them that may control the color of *R. delavayi* flowers. From this Figure, it can be seen that *Rhdel02G0041700*, *Rhdel03G0013600*, and *Rhdel03G0341200* may be the key genes controlling *R. delavayi* flower color.

## 4. Discussion

bHLH transcription factors play a pivotal role in plant growth, development, and responses to abiotic stresses [14]. The number of bHLH gene family members varies considerably across species. For example, 208 members have been identified in maize, 95 in red raspberry [30], 183 in rice [31], and 81 in Nepal goldenrod [32]. However, research on the bHLH gene family in *R. delavayi* remains limited. Previous studies identified 116 bHLH genes in *R. delavayi*, slightly fewer than the 145 identified in this study. Notably, three bHLH genes were located on contig52. Analysis of the physicochemical properties of these gene family members revealed a broad isoelectric point range (4.22–10.87), suggesting adaptability to a wide pH spectrum.

The 145 members of the *R. delavayi* bHLH gene family are unevenly distributed across 13 chromosomes. Chromosomes 9 and 13 contain the fewest bHLH genes (7 each), accounting for 4.8% of the total gene number, while chromosome 8 contains the most, with 19 genes, representing 13.1% of the total. This distribution suggests that the number of bHLH genes on a chromosome is not correlated with chromosome size. Phylogenetic analysis further indicates that bHLH genes in *R. delavayi* are relatively evenly distributed across chromosomes, with no apparent chromosomal preferences.

Conserved motif analysis of the *R. delavayi* bHLH transcription factors revealed a highly conserved motif structure. Motif 1 and Motif 2 are present in nearly all *R. delavayi* bHLH transcription factors, consistent with previous studies [32]. Subcellular localization predictions suggest that the vast majority of these bHLH genes are localized in the nucleus, indicating that their primary function occurs within the nucleus.

Cis-acting element analysis of the promoters of the *R. delavayi* bHLH gene family revealed that these elements are mainly associated with processes such as growth and development, hormone responses, light responses, and stress responses. Notably, cis-acting elements related to light response are the most abundant, suggesting that *R. delavayi* bHLH genes play a significant role in plant light response processes [33], in agreement with the findings of this study. Additionally, cis-acting elements were predominantly distributed within core promoter regions near the transcription start site (−30), particularly related to light response.

bHLH transcription factors are key regulators of anthocyanin biosynthesis. Studies have shown that bHLH plays a critical role in anthocyanin glycoside synthesis in chrysanthemum. Heterologous expression of the bHLH homolog *Lc* has been found to increase anthocyanin glycoside content [34]. In herbaceous peony, the *PqbHLH1* gene positively regulates anthocyanin and flavonol synthesis in leaf tissues by modulating the expression of genes related to anthocyanin biosynthesis [35]. In Sichuan garlic orchid, *PlbHLH* and *PlWD40* are thought to regulate *PlFLS* expression in flowers of three different colors, potentially driving color variation through the formation of *PlMYB10/PlbHLH20/PlWD40-1* or *PlMYB10/PlbHLH26/PlWD40-1* complexes, in association with *PLMYB10* [36]. In green stems of *Panax ginseng var. notoginseng*, the expression levels of bHLH, MYB, and WD40 transcripts are positively correlated with total anthocyanin content (TAC) [15]. Similarly, in higher plants, anthocyanin biosynthesis is commonly regulated by bHLH, MYB, and WD40 complexes [37]. As an ornamental tree, flower color is a key aesthetic trait, with red being the predominant color. The MYB, bHLH, and WD40 genes are the main regulators of flower color in plants [38]. Currently, research on plant flower color has primarily focused on MYB genes, with relatively little attention given to the bHLH gene family [39,40]. In the variety ‘Endless Summer’, transcriptome data and bioinformatics analysis identified 11 MYB transcription factors, 2 bHLH transcription factors, and 1 WD40 transcription factor potentially involved in anthocyanin biosynthesis [41].

In *Dianthus annuus*, the non-IIIf subfamily members *CrbHLH59* and *CrbHLH71* were highly expressed in the cultivar ‘Kid’s Face’. *CrbHLH59* was negatively correlated with cornflower pigment glycosides, while *CrbHLH71* was positively correlated with proanthocyanidins. It is hypothesized that the high expression of *CrbHLH59* and *CrbHLH71* in ‘Kid’s Face’ decreased the synthesis of red cornflower pigment glycosides and increased the accumulation of colorless proanthocyanidins, resulting in the white color of the cultivar [42]. Additionally, the *ZeGL3* gene, a member of the IIIf subfamily in *Zinnia elegans*, has been implicated in the regulation of anthocyanin glycoside metabolism [43].

Transcriptome expression analysis of the *DdbHLH* gene family in *Docynia delavayi* (Yunnan rhea) revealed that *DdbHLH68* and *DdbHLH150* were significantly upregulated in red fruit skins, suggesting that these two genes play important roles in anthocyanin accumulation [44]. In this study, differential gene expression pattern analysis identified *Rhdel02G0041700*, *Rhdel03G0013600*, and *Rhdel03G0341200* as potential key genes controlling flower color in *R. delavayi*. However, their specific functions require further experimental verification.

## 5. Conclusions

Flower color is a significant ornamental trait in *Rhododendron* and serves as an adaptive feature related to pollinators and the natural environment. The flower pigments in *Rhododendron* primarily consist of anthocyanin glycosides and flavonols. The types, concentrations, and associated genes of these pigments critically influence flower color formation. Understanding the composition of flower pigments and their biosynthetic pathways is essential for breeding *Rhododendron* varieties with desirable flower colors. Red flowers are particularly abundant in *Rhododendron*, with ideal breeding parents such as *R. delavayi*, *R. haematodes*, and *R. fortunei* predominantly found in China. Flower color significantly impacts future *Rhododendron* breeding efforts.

In this study, 145 bHLH transcription factor family members were identified in *R. delavayi* through whole-genome analysis. bHLH transcription factors are crucial plant regulatory elements involved in gene expression modulation. This research explores the complex characteristics, regulatory mechanisms, and roles of bHLH transcription factors in flower color regulation in *R. delavayi*, providing a robust foundation for further investigation into the mechanisms underlying flower color regulation in this species.

## Data Availability

The data presented in this study are openly available in the genomic sequence, and GFF annotation file of R. delavayi are derived from the Rhododendron Plant Genomic Database (http://bioinfor.kib.ac.cn/RPGD/index.html) the original RNA-Seq data related to flower color from the NCBI database with the data ID PRJNA907866.

## Supplementary Materials

The following supporting information can be downloaded at: https://www.mdpi.com/article/10.3390/genes15101256/s1, Supplementary Table S1.
